# DyMnO_3_: Synthesis, Characterization and Evaluation of Its Photocatalytic Activity in the Visible Spectrum

**DOI:** 10.3390/ma16247666

**Published:** 2023-12-15

**Authors:** Miguel Ángel López-Álvarez, Pedro Ortega-Gudiño, Jorge Manuel Silva-Jara, Jazmín Guadalupe Silva-Galindo, Arturo Barrera-Rodríguez, José Eduardo Casillas-García, Israel Ceja-Andrade, Jesús Alonso Guerrero-de León, Carlos Alberto López-de Alba

**Affiliations:** 1 Departamento de Ingeniería Mecánica, Centro Universitario de Ciencias Exactas e Ingenierías, Universidad de Guadalajara, Blvd. Marcelino García Barragán 1421, Guadalajara 44430, Jalisco, Mexico; alonso.guerrero@academicos.udg.mx (J.A.G.-d.L.); carlos.ldealba@academicos.udg.mx (C.A.L.-d.A.); 2 Departamento de Ingeniería Química, Centro Universitario de Ciencias Exactas e Ingenierías, Universidad de Guadalajara, Blvd. Marcelino García Barragán 1421, Guadalajara 44430, Jalisco, Mexico; 3 Departamento de Farmacobiología, Centro Universitario de Ciencias Exactas e Ingenierías, Universidad de Guadalajara, Blvd. Marcelino García Barragán 1421, Guadalajara 44430, Jalisco, Mexico; jorge.silva@academicos.udg.mx (J.M.S.-J.); jazmin.silva@alumnos.udg.mx (J.G.S.-G.); 4 Centro de Investigación en Nanocatálisis Ambiental y Energías Limpias CUCIENEGA, Universidad de Guadalajara, Av. Universidad 1115, Ocotlán 47820, Jalisco, Mexico; arturo.barrera@academicos.udg.mx; 5 Departamento de Ciencias Tecnológicas, Centro Universitario de la Ciénega (CUCIENEGA), Universidad de Guadalajara, Av. Universidad 1115, Ocotlán 47820, Jalisco, Mexico; jose.casillas2569@academicos.udg.mx; 6 Departamento de Física, Centro Universitario de Ciencias Exactas e Ingenierías, Universidad de Guadalajara, Blvd. Marcelino García Barragán 1421, Guadalajara 44430, Jalisco, Mexico; israel.ceja@academicos.udg.mx

**Keywords:** DyMnO_3_, photocatalysis, malachite green dye, visible-light photocatalyst

## Abstract

DyMnO_3_ is a p-type semiconductor oxide with two crystal systems, orthorhombic and hexagonal. This material highlights its ferroelectric and ferromagnetic properties, which have been the subject of numerous studies. Nevertheless, its photocatalytic activity has been less explored. In this work, the photocatalytic activity of DyMnO_3_ is evaluated through the photodegradation of MG dye. For the synthesis of this oxide, a novel and effective method was used: polymer-decomposition. The synthesized powders contain an orthorhombic phase, with a range of absorbances from 300 to 500 nm and a band gap energy of 2.4 eV. It is also highlighted that, when using this synthesis method, some of the main diffraction lines related to the orthorhombic phase appear at 100 °C. Regarding its photocatalytic activity, it was evaluated under visible light (λ = 405 nm), reaching a photodegradation of approximately 88% in a period of 30 min. Photocurrent tests reveal a charge carrier separation ($e^{-},h^{+}$) at a 405 nm wavelength. The main reactive oxygen species (ROS) involved in the photodegradation process were radicals, ${OH}^{•},$ and photo-holes ($h^{+}$). These results stand out because it is the first time that the photodegradation capability of this oxide in the visible spectrum has been evaluated.

## 1. Introduction

Today’s landscape is contaminated with heavy metals and inorganic and organic compounds in seas, rivers, and lakes. Organic contaminants are the dyes used substantially in the textile, paper, and pharmaceutical industries, and in our environment, they can be found in products from plastics to toys [1]. Among the dyes that stand out for their toxic effect are azo dyes. They are characterized by the presence of one or more azo groups (-N=N-) linked to aromatic rings such as benzene and naphthalene. They are normally resistant to biodegradation. These dyes are widely used in the textile, leather, food, cosmetics, and paper products industries due to their fastness and variety of colors compared to natural ones. The annual global production of azo dyes is estimated at around one million tons, and more than 2000 azo dyes are currently used [2]. Because many of them are soluble in water and are related to various carcinogenic processes, different physicochemical techniques have been proposed for their degradation, such as adsorption, coagulation, electrodeionization, precipitation, photolysis, or membrane filtration [3]. Sometimes, these techniques are inefficient, expensive, and consume a large amount of energy. Therefore, photocatalysis has been proposed as an alternative technique for the degradation of azo dyes [4].

Of the group of azo dyes, one of the most representative is malachite green (MG) dye. MG is a cationic dye used as a fungicide and antiseptic in aquaculture and the industries of textile and paper [5]. However, MG dye represents a risk to human health due to its negative effects on the immune and reproductive systems, as well as its potential genotoxicity and carcinogenic properties [6]. Thus, diverse oxides have been used as photocatalysts for the degradation of MG dye, including: Fe_2_O_3_, TiO_2_, ZnO, CeO_2_, WO_3_, V_2_O_5_, Cu_2_O, CuO, NiO, Mn_3_O_4_, Ag_2_O, and CuMn_2_O_4_ [4]. Furthermore, the photodegradation of MG dye with an yttrium perovskite photocatalyzed in the visible spectrum was recently observed [7]. Nevertheless, there are other groups of oxides that have been used in the photodegradation of azo dyes at wavelengths in the visible spectrum. These are oxides with a perovskite-type structure (ABO_3_) (where A is a rare earth or alkaline metal, B is a transition metal, and O is oxygen), such as: XTaO_3_ (X = Na, K, Ag, Li), XNbO_3_ (X = Na, K, Ag, Cu), XTiO_3_ (X = Sr, Ba, Ca, Ni), XFeO_3_ (X = Y, La, Bi), and AgVaO_3_ [8]. The reasons why oxides with a perovskite-type structure are considered potential candidates for the photodegradation of organic dyes are the following: they are formed from a wide variety of compositional and constituent elements; their valency, stoichiometry, and vacancy can be varied widely; and there is much information on their physical and solid-state chemical properties [9].

On the other hand, the oxides with a perovskite-type structure, whose photocatalytic activity has been the subject of recent research, are the rare earth manganites. Of this group of perovskites, DyMnO_3_ stands out, mainly, for its ferroelectric, ferromagnetic [10,11,12], and magnetocaloric properties [13,14], which have contributed to its technological applications [15,16]. However, its optical applications (for example, its use as a photocatalyst) have been little explored.

Considering the above, the synthesis of DyMnO_3_ and its use as a photocatalyst in the degradation of MG dye are proposed in this work. Furthermore, its photocatalytic activity will be evaluated at a wavelength in the visible spectrum (*λ* = 405 nm). Regarding its characterization, this will be carried out by XRD, TEM, UV-Vis, and XPS spectroscopies. It should be noted that it is the first time that the photocatalytic activity of DyMnO_3_ has been evaluated at a wavelength of the visible spectrum, as well as the first time that the polymer-decomposition method has been used for the synthesis of this perovskite.

## 2. Materials and Methods

### 2.1. Synthesis

#### 2.1.1. Precursor Optimization

In the synthesis, the concentrations of dysprosium nitrate hydrate (Sigma Aldrich, Saint Louis, MO, USA, 99.9%) and manganese nitrate tetrahydrate (Sigma Aldrich, 97%) were 1.388 and 1 g, respectively (The concentration would be 4 × 10^−3^ M for each reagent). Both were mixed simultaneously and diluted with 20 mL of bi-distilled water. Subsequently, 0.200 g of polyvinyl alcohol (PVA) (Sigma Aldrich, 99.8%, Mw = 89,000–98,000), diluted in 20 mL of bi-distilled water, was added to the solution containing the metal cations. The final mixture was stirred moderately for 30 min and placed in a drying oven for 1 h. This caused almost 70% of the water volume to evaporate, and a viscous solution was obtained. Finally, this solution was placed in a conventional microwave oven for 2 min. During that period, an exothermic reaction occurred, releasing an abundant amount of gases. The product of this reaction was a porous-looking black powder.

#### 2.1.2. Temperature Optimization

The precursor powder obtained from the PVA decomposition reaction was calcined at 800, 1000, and 1200 °C. These temperatures were selected because, in previous research, it was observed that, from 800 °C onwards, the orthorhombic phase of this material begins to be obtained. All calcinations were for 3 h.

### 2.2. Characterization of DyMnO_3_ Powders

The crystallinity evaluation of DyMnO_3_ powders was carried out using an Empyrean diffractometer (PANalytical, Westborough, MA, USA) with CuK1 radiation. The morphology analysis was performed using transmission electron microscopy (TEM) with a Jeol JEM1010 microscope (Tokyo, Japan). The absorbance spectrum of the dysprosium manganate powders was obtained using a UV-Vis spectrophotometer (Cary 100 Agilent Technologies, Santa Clara, CA, USA).

### 2.3. Photocatalytic Activity

To evaluate the photodegradation of the malachite green dye (MG) using DyMnO_3_ as a photocatalyst, two solutions with 40 mL of this dye at a concentration of 1.5 × 10^−5^ M were prepared. To one solution, 20 mg of DyMnO_3_ was added, while 40 mg was added to the other. Both solutions were exposed to visible light (*λ* = 405 nm) for 180 min. An LED (Light Emitting Diode) with an optical irradiance (E_e_) of 100 mW/cm^2^ was used as the light source. Then, aliquots were extracted at different intervals and analyzed using a NanoDrop 2000 spectrophotometer (Thermo Scientific, Waltham, MA, USA). The degradation percentage was estimated using Equation (1).

(1) $$Degradation=\frac{A_{0}-A}{A_{0}}=\frac{C_{0}-C}{C_{0}}\times 100$$

where A_0_ and A correspond to the solutions’ absorbance values before and after exposure to visible light, respectively. Similarly, C_0_ and C represent the concentrations of the solutions before and after irradiation, respectively.

### 2.4. Transient Photocurrent Tests

To carry out this test, a pellet with a diameter of 1.2 cm and a thickness of 2 mm of DyMnO_3_ was fabricated. The pellet was sintered at 600 °C for 12 h. Subsequently, it was placed on two NiAg foils with a thickness of approximately 1 mm. These foils served as electrodes, applying a direct voltage (DC) of 5 V to the pellet. Then, the pellet was exposed to visible light (*λ* = 405 nm) using an LED with the same optical irradiance as in the photocatalysis experiments. Finally, the photocurrent measurements were obtained using a DMM 6500 multimeter (Keithley, Portland, OR, USA) under the conditions described above.

The emission spectrum of the LED was obtained using a CCS200 Spectrometer (Thorlabs, NJ, USA).

## 3. Results and Discussion

### 3.1. X-ray Diffraction

**Figure 1:**
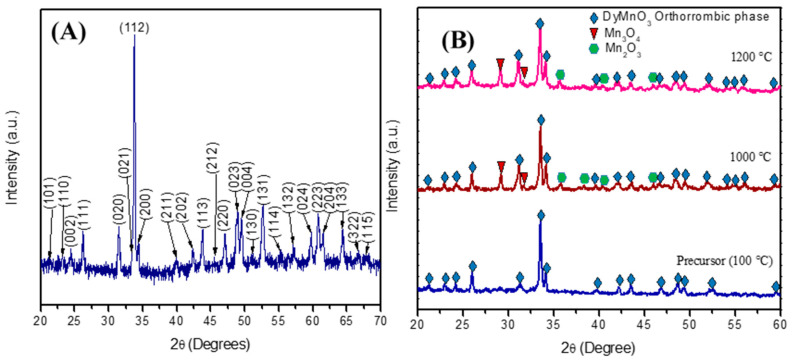
(**A**) Diffractograms of precursor powder calcined at 800 °C and (**B**) its calcinations a 100, 1000, and 1200 °C.

Figure 1 shows the diffraction pattern of the calcined precursor powder at 800 °C. As can be observed, each diffraction line was identified and associated with the orthorhombic phase of DyMnO_3_ (JCPDF #25-0330). It is worth noting that no diffraction lines associated with the hexagonal phase or with the oxides Dy_2_O_3_, MnO_2_, or Mn_2_O_3_ were observed. Moreover, when this synthesis method is compared with some of those used for obtaining the orthorhombic phase, it was observed that the polymer-decomposition method achieves this phase at a lower temperature and without the use of acidic or organometallic reagents [17,18].

In order to evaluate the crystalline evolution and propose a formation mechanism for DyMnO_3_, diffraction patterns of the precursor powder and its calcinations at 1000 and 1200 °C were analyzed by XRD. The diffractogram of the precursor powder calcined at 100 °C shows some of the main diffraction lines related to the orthorhombic phase. According to this result, it is observed that the synthesis method used in this work promotes obtaining the orthorhombic phase, even when using a relatively low calcination temperature. On the other hand, the XRD patterns of the calcinations of the precursor powder at 1000 °C and 1200 °C show diffraction lines with greater intensities. Furthermore, more diffraction peaks associated with the orthorhombic phase appear after the calcinations. However, these calcination temperatures also promote the formation of impurities associated with manganese, particularly the presence of the oxides Mn_2_O_3_ (JCPDF #41-1442) and Mn_3_O_4_ (JCPDF #24-0734).

Based on the XRD patterns of the calcinations carried out on the precursor (Figure 1B), the following reaction to obtain DyMnO_3_ is proposed:

(2) $$Dy{\left(NO_{3}\right)}_{3}\times xH_{2}O+Mn{\left(NO_{3}\right)}_{2}\times 4H_{2}O+{\left(CH_{2}-CHOH\right)}_{n}+H_{2}O\underset{800{}^{\circ}c}{\to }DyMnO_{3}+\alpha \left(NO_{x}\right)+{\beta (H}_{2}O)+\varepsilon (CO_{2})$$

According to Equation (2), the mechanism to obtain the orthorhombic phase can be described as follows: The metal cations are bound to the hydroxyl groups of the polymer. This causes the cations to stabilize in the polymer structure via interactions with the hydroxyl groups [19]. Then, a precipitation process does not occur. When the solution obtained is placed inside a conventional microwave oven, a polymer-decomposition reaction at approximately 100 °C occurs. During the reaction, the manganese cation is oxidized and the chemical structure of PVA is decomposed. Thus, an abundant amount of gases is released, as shown by Equation (2). At the end of the reaction, the metal cations adopt the architecture of a polymer network.

### 3.2. Transmission Electron Microscopy

**Figure 2:**
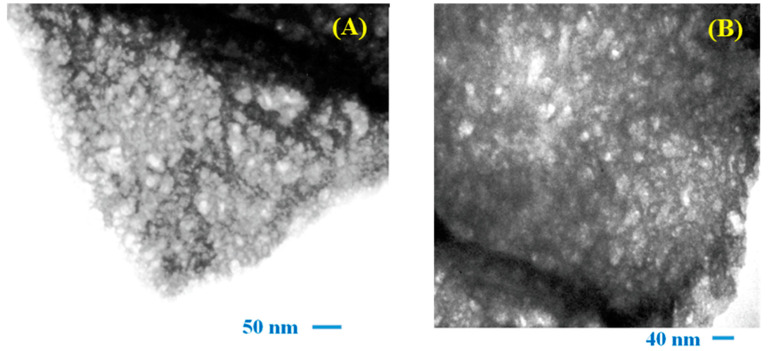
TEM micrographs of DyMnO_3_ powders at (**A**) 80,000 and (**B**) 100,000 magnifications.

Figure 2A,B show the TEM micrographs of the DyMnO_3_ powders. In both images, flake-shaped nanostructures with porosity can be observed. Notably, the presence of porosity may be related to the release of NO_x_ gases, which are produced during the thermal decomposition of the manganese and dysprosium nitrates used in the synthesis process. It should be mentioned that this type of morphology has already been observed in other oxides synthesized using the polymer-decomposition method [19].

Based on other research, it is known that micro- or nanostructures containing porosity can contribute to the photodegradation of dyes because they promote their absorption and, also, interactions between reactive oxygen species (ROS) and the dye molecule occur more efficiently [20,21].

### 3.3. Porosity Analysis

**Figure 3:**
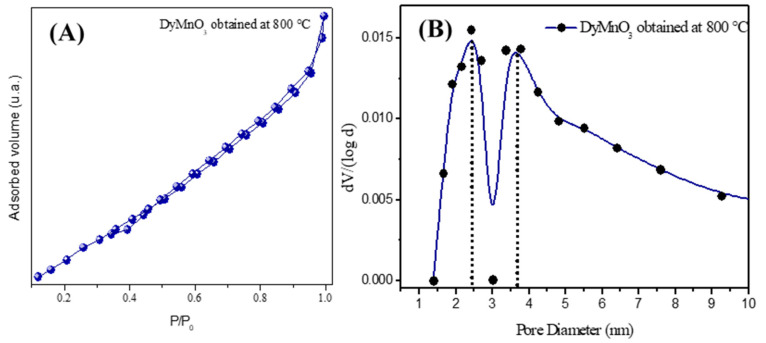
(**A**) Nitrogen adsorption*—*desorption isotherm and (**B**) pore size distribution of DyMnO_3_. The blue circles correspond to the data acquired from the experiment. The black circles dotted line represents two modal peaks corresponding to two more frequent pore sizes.

Figure 3 shows the nitrogen adsorption*—*desorption isotherm of DyMnO_3_ obtained at 800 °C. This isotherm can be considered II-type, which is related to macroporous material [22]. The specific surface area (SBET) was 7.3 m^2^ g*^−^*^1^, which is similar to that corresponding to lanthanide oxides. Its porosity is corroborated by its total specific pore volume (0.0113 cm^3^ g*^−^*^1^) and its micropore volume (0.000128 cm^3^ g*^−^*^1^) calculated by the BJH and DR methods [22], respectively. These results are summarized in Table 1.

The pore diameter of DyMnO_3_ calculated with the BJH method was 2.2 nm, and it is at the limit of the mesopore range according to the IUPAC classification [23]. Its pore size distribution is bimodal since it exhibits two modal peaks corresponding to two more frequent pore sizes at 2.4 and 3.7 nm.

### 3.4. UV-Vis Spectroscopy

**Figure 4:**
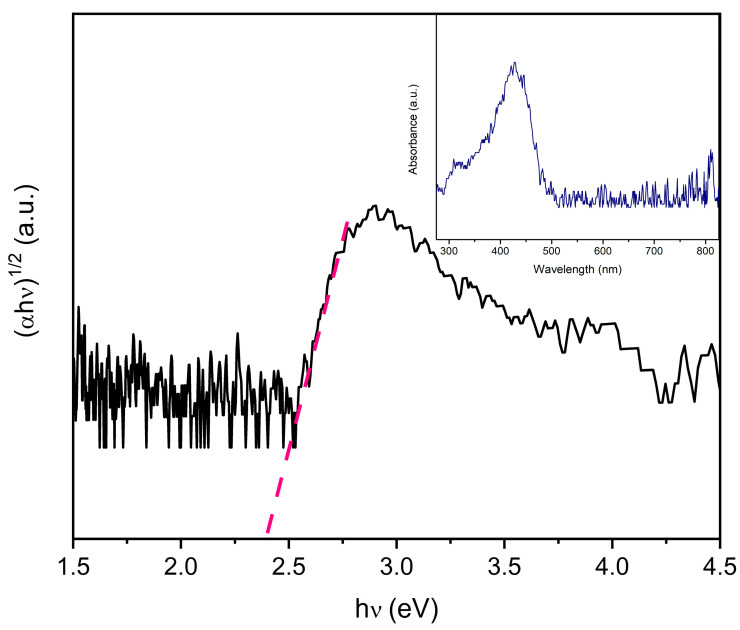
Tauc plot of DyMnO_3_. Within the graph is the absorption spectrum of this oxide. The pink line represent the intersection with the *x*-axis indicates the band gap energy. The blue lines correspond to the absorbance spectrum of DyMnO_3_, and the black lines are the graphic results of the Tauc-plot.

Figure 4 shows the graph of ${\left(\alpha h\nu \right)}^{n}\;vs.\;h\nu$ (Tauc plot), where α represents the absorption coefficient, h is the Planck constant, and υ is the frequency of the light. The exponent, n, value is associated with the type of electronic transition the material exhibits, with n = 2 for direct and n = 1/2 for indirect transitions. For DyMnO_3_, the value is n = ½ [24]. In the graph, the intersection of the dashed line with the x-axis indicates the band gap energy (*E_g_*). In this case, the approximate value of *E_g_* is 2.4 eV. Based on other research, it is known that the valence band (VB) of rare-earth manganates (such as DyMnO_3_) consists of O 2p and lanthanide 4f orbitals, while the conduction band (CB) consists of Mn 3d orbitals [24].

Within the Tauc plot, the absorbance spectrum of the DyMnO_3_ powders is also shown. As can be observed, this oxide exhibits an absorbance range that extends from 300 nm to 500 nm, making it a potential candidate for evaluating its photocatalytic activity at wavelengths in the visible spectrum.

By applying Equation (3) to the band gap energy obtained in the Tauc plot, it is possible to estimate the minimum wavelength required to promote electrons from the VB to migrate to the CB. In this case, the value obtained is 516 nm. Based on this result, DyMnO_3_ can be considered a potential candidate to be used as a photocatalyst at wavelengths in the visible spectrum.

(3) $$\lambda \;(nm)=\frac{1240}{E_{g}\;(eV)}$$

### 3.5. Transient Photocurrent Measurements

**Figure 5:**
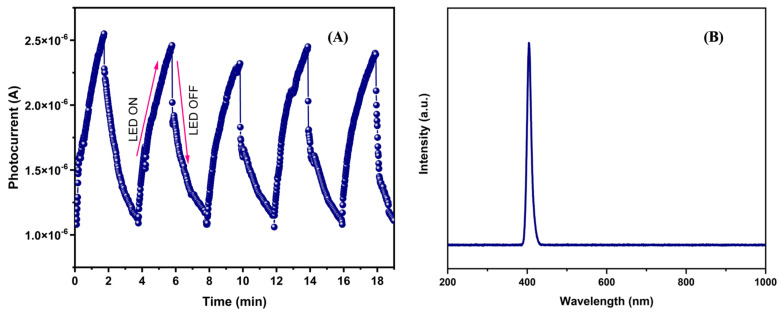
(**A**) Graphical results of transient photocurrent measurements using a wavelength in the visible spectrum (λ = 405 nm) at an optical irradiance of 100 mW/cm^2^. (**B**) Emission spectrum of LED.

Figure 5A shows the photocurrent vs. time graph obtained from transient photocurrent measurements. During the light exposure period, the electric current in the material increases, while in the absence of light, it decreases. The increase in photocurrent was approximately 2.44 × $10^{-6}$ A. This reveals that the incident photons (with energy hν = 3.1 eV) promote the creation of electron–hole pairs ($e^{-},h^{+}$) on the surface of this material, preventing their recombination. Subsequently, the applied voltage contributes to the mobility of the charge carriers, generating an electric current.

Additionally, Figure 5B shows the emission spectrum of the LED used in the transient photocurrent tests and photocatalysis experiments. As observed, the light emitted by the LED has a wavelength of 405 nm. Furthermore, it can be considered monochromatic because no contributions from other wavelengths that could influence the photocatalytic activity of this oxide are detected.

### 3.6. Degradation of Malachite Green (MG) Dye

**Figure 6:**
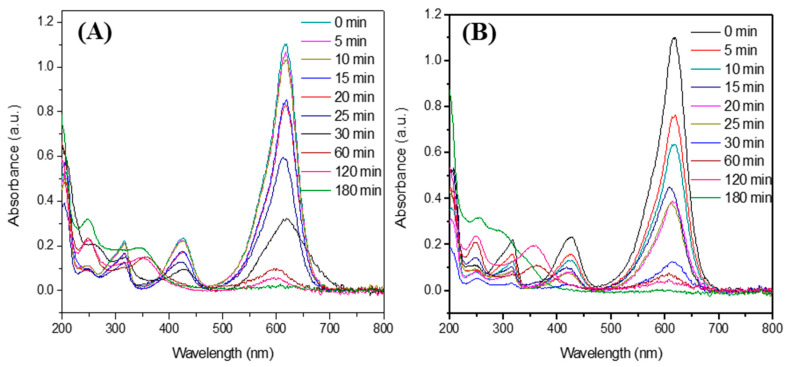
UV-Vis absorbance spectra obtained during the degradation of the MG dye using (**A**) 20 and (**B**) 40 mg of DyMnO_3_ as a photocatalyst.

**Figure 7:**
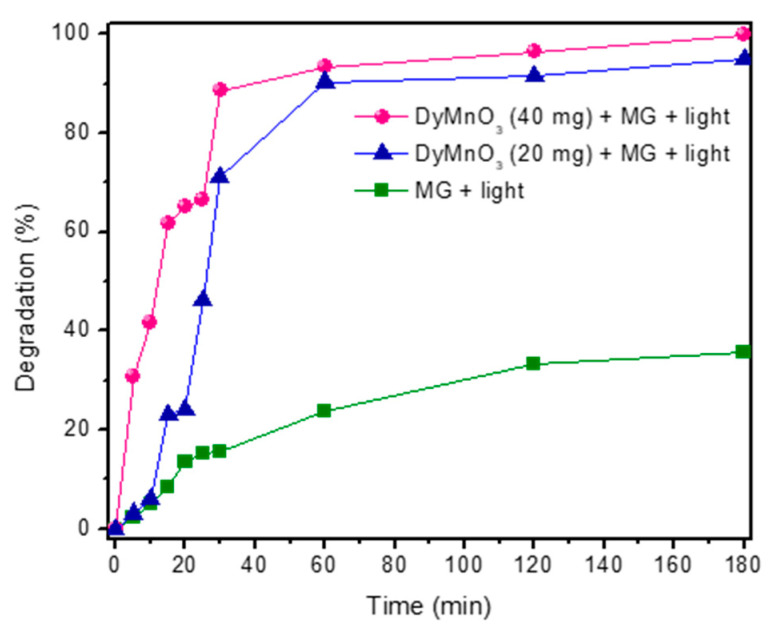
Percentages of photocatalytic degradation of MG dye using 20 and 40 mg of DyMnO_3_. The peak of highest absorbance located at 617 (associated with N-methyl groups) was selected as a reference to estimate the percentage of degradation.

Figure 6A,B show the absorbance spectra of the aliquots extracted from MG solutions containing 20 mg and 40 mg of DyMnO_3_, respectively. A significant decrease in the absorbance peak located at approximately 617 nm can be observed. Specifically, it is observed that 40 mg of this oxide can promote the photodegradation of the dye by 30.8 and 88.6% in 5 and 30 min, respectively (Figure 7). Meanwhile, with 20 mg, the percentages achieved in the same time intervals are 3.1 and 71.1%, respectively. This increase in photocatalytic activity can be attributed to increasing the amount of DyMnO_3_.

During the photodegradation process, the absorbance spectra of the MG dye show a slight blue shift of the major absorbance peak, from 617 to 598 nm. This shift has been observed in other research and was related to a process of N-demethylation that occurred in the degradation of the dye [25]. Additionally, a decrease in the intensity of the absorbance peaks located at 425 and 315 nm is also observed, indicating that the chromophore groups of the MG dye have been destroyed. These results reveal that reactive oxygen species (ROS) attack the C-N bonds of the MG dye, causing its degradation.

To evaluate the contribution of the light (λ = 405 nm, *E_e_* = 100 mW/cm^2^) to the photodegradation of this dye, 40 mL of the MG dye was exposed to this wavelength of light without adding DyMnO_3_. The results revealed that after 180 min, a photodegradation of 35.6% occurred. However, this value can be considered negligible compared to those obtained using 20 and 40 mg of photocatalyst.

Due to the conditions in which these photodegradation experiments were carried out (*E_e_* = 100 mW/cm^2^, λ = 405 nm), it is difficult to compare the photocatalytic efficiency of DyMnO_3_ with other oxides used as photocatalysts. However, Table 2 shows some photocatalyst oxides used in the degradation of MG dye at an optical irradiance of 100 mW/cm^2^.

The first three have a perovskite-type structure. However, in all cases, DyMnO_3_ shows greater efficiency in degrading this dye.

**Figure 8:**
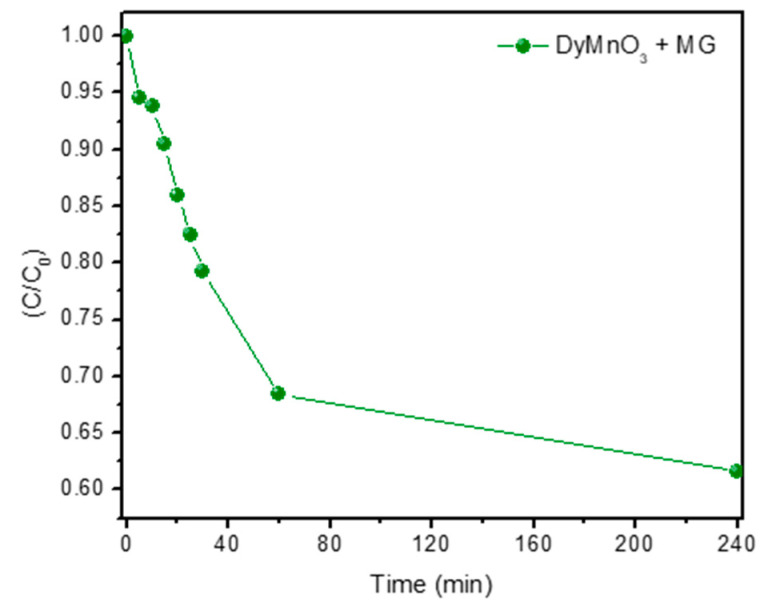
Graphic results of the DyMnO_3_ absorption tests on the MG dye.

Additionally, the contribution of DyMnO_3_ absorption to the degradation process of the MG dye was evaluated. In this test, 40 mg of the photocatalyst powder was added to a solution containing 40 mL of the MG dye. Figure 8 shows the graphic results. As can be observed, the greatest degradation occurs in the 60 min period, at approximately 32%. After 240 min, the dye degrades by approximately 40%. These results confirm that the degradation of the dye can be attributed mainly to the formation of ROS rather than to the absorption process that occurred on the surface of the photocatalyst.

#### 3.6.1. Photodegradation Kinetics

To identify and associate a kinetic model with the photocatalytic degradation process of MG dye using DyMnO_3_ as a photocatalyst, the pseudo-first-order kinetic model was selected as the kinetic model (Equation (4)).

(4) $$In\left(\frac{C_{0}}{C}\right)=kt$$

where *C*_0_ and *C* represent the concentration values of the solutions at the beginning and after a time t of light exposure, respectively; the photochemical degradation rate is represented by *k*.

**Figure 9:**
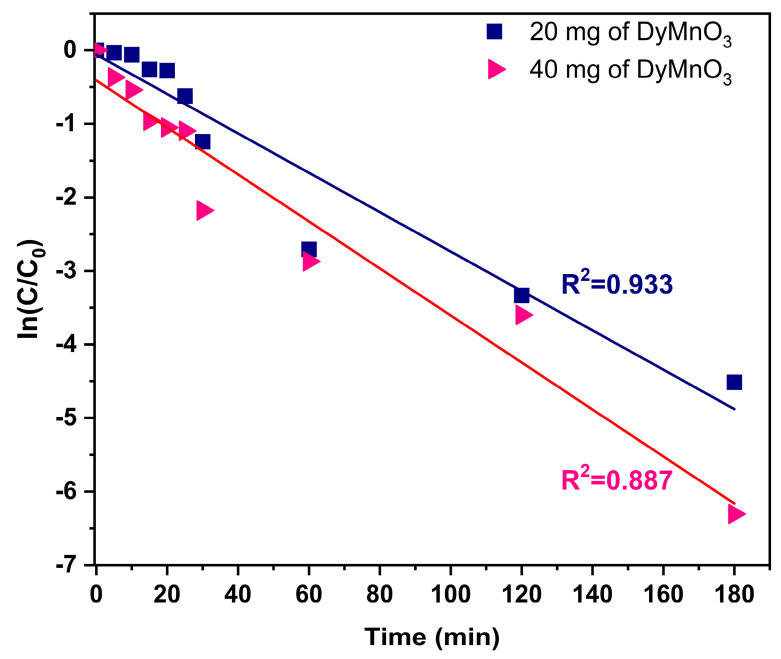
Linear fit corresponds to the pseudo-first-order kinetic model using 20 and 40 mg of DyMnO_3_.

Figure 9 shows the graphical results obtained using the pseudo-first-order kinetic model, with the absorbance values recorded in the spectra of Figure 5. The straight line in each graph corresponds to the linear fit applied to the results after using the pseudo-first-order kinetic model. As can be seen, the correlation coefficients (R^2^) were 0.93 (20 mg) and 0.88 (40 mg). These results indicate that the absorbance values recorded during the photocatalytic degradation process of the MG dye closely relate to the proposed kinetic model. It should be mentioned that other kinetic models were also used, such as: the zero-order (R^2^ < 0.50), second-order (R^2^ < 0.70), pseudo-second-order (R^2^ < 0.70), parabolic diffusion (R^2^ < 0.70) and modified Freundlich models (R^2^ < 0.60). However, their correlation coefficients were less than 0.70. Additionally, the photocatalytic degradation rate (k) was also obtained. The apparent values for k were 0.020 min^−1^ (20 mg) and 0.031 min^−1^ (40 mg).

Although there are many studies based on the photodegradation of MG dye with photocatalyzing oxides, only some have reported the estimated value of the k constant. Among the values of the constant k reported for Cu/ZnO [29], ZnFeO_4_ [30], and ZnO [31] were 10$\times 10^{-3}$, 7.1 $\times 10^{-3}$ , and 20.4 $\times 10^{-3}$, respectively. However, it is difficult to compare the values of k obtained in this research with those recorded previously. This is because the values obtained in the other works were in different concentrations, and, in addition, their linear fits present an R^2^ close to 0.99.

It is evident that the proposed kinetic model (pseudo-first-order kinetics) does not completely fit the photodegradation process of the MG dye with DyMnO_3_. Furthermore, when the zero-order, second-order, pseudo-second-order, parabolic diffusion, and modified Freundlich models were applied in this photodegradation process, they showed a correlation coefficient of less than 0.70. One of the possible causes associated with this chemical behavior may be the following: In most kinetic models, the absorption rate is greater than the photodegradation rate. However, in this case, the photodegradation rate exceeds the absorption rate.

#### 3.6.2. Recycling Tests and pH Influence

**Figure 10:**
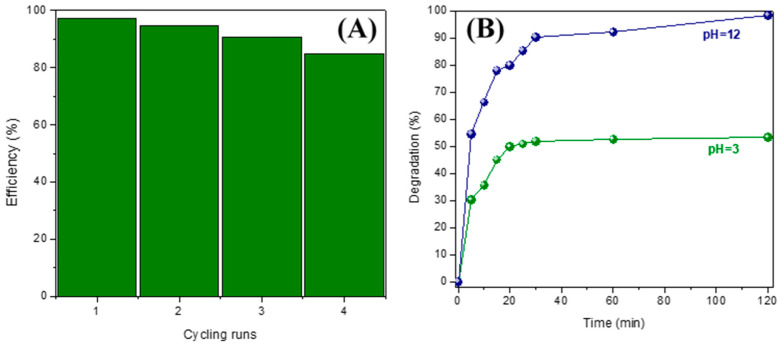
(**A**) Reusability of DyMnO_3_ photocatalyst for MG dye degradation. (**B**) Influence of pH on the degradation of the MG dye using DyMnO_3_ as a photocatalyst.

With the purpose of evaluating the reuse of DyMnO_3_ as a photocatalyst, recycling tests were carried out. In four cycles, a decrease in the degradation rate was observed (Figure 10A). It is possible that fragments of the dye molecule are absorbed on the surface of the photocatalyst, causing a decrease in its degradation rate.

Additionally, the influence of pH on the surface of the photocatalyst was evaluated. For this, two solutions with pH = 3 and pH = 12 were prepared. Then, 40 mg of DyMnO_3_ was added to each solution and exposed to visible light (λ = 405 nm; *E_e_* = 100 mW/cm^2^). Figure 10B shows the graphical results. In 120 min, degradations of approximately 53% (pH = 3) and 98% (pH = 12) were achieved. Although it is difficult to interpret the influence of pH on the photocatalytic reaction due to its multiple roles, a possible interpretation of this result could be the following: by increasing the pH, the surface charge of the photocatalyst would be negative. This may be possible because DyMnO_3_ is a p-type semiconductor; therefore, it electrostatically attracts negative charges. This causes the dye to be absorbed more effectively on the surface of the photocatalyst and its degradation to occur more effectively.

#### 3.6.3. XPS Analysis

**Figure 11:**
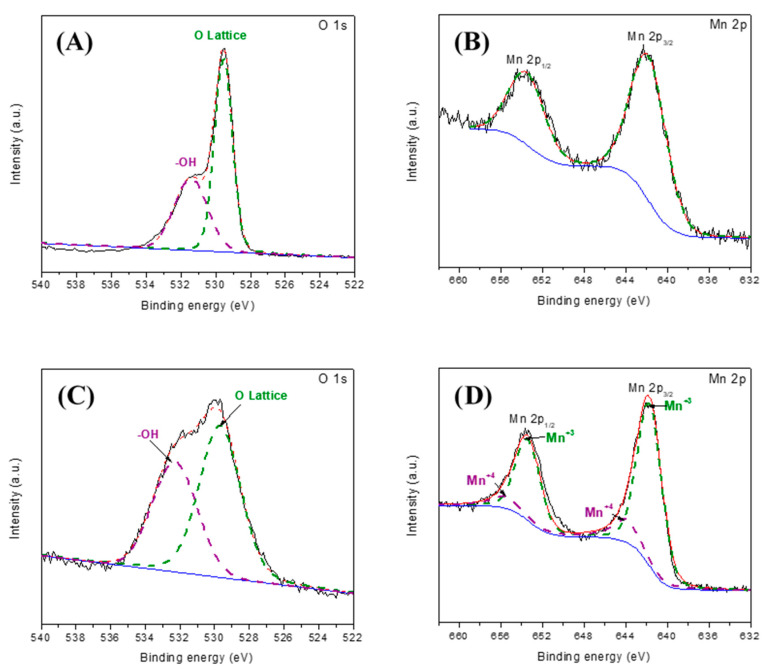
XPS spectrum of the O 1s and Mn 2p core levels before (**A**,**B**) and after (**C**,**D**) DyMnO_3_ was used as a photocatalyst, respectively. The dashed lines represent the deconvolutions.

To identify and associate the chemical species present on the DyMnO_3_ powder surface, the O 1s and Mn 2p core levels (Figure 11) were analyzed through XPS.

Before being used as a photocatalyst, the O 1s XPS spectrum (Figure 11A) was deconvoluted into two Gaussians centered at approximately 529.5 and 531.4 eV. The former was associated with Dy-O and Mn-O bonds, while the latter was attributed to the presence of hydroxyl groups (-OH) [32]. Additionally, the area of each Gaussian curve was quantified: 60.24% for the curve associated with the Dy-O and Mn-O bonds (O Lattice) and 39.76% for the one associated with -OH. After being used as a photocatalyst, the new O 1s spectrum (Figure 11C) was also deconvoluted. The results show two Gaussians associated with the same chemical species described previously. However, the area covered by each was 54.04% (O Lattice) and 45.96% (-OH). Comparing these results with those obtained before being used as a photocatalyst, an approximately 6.2% increase in -OH was quantified.

The XPS spectrum of Mn 2p was also analyzed before DyMnO_3_ was used as a photocatalyst (Figure 11B). Two peaks located at approximately at 642 eV (Mn 2$p^{3/2}$) and 653.7 eV (Mn 2$p^{1/2}$) appear in its spectrum, which are associated with the +3 oxidation state of manganese (${Mn}^{+3}$) [30]. However, after being used as a photocatalyst (Figure 11D), the Mn 2p spectrum was deconvolved. The Gaussians located at 641.9 and 653.5 eV were associated with the +3 state of manganese (${Mn}^{+3}$), while those located at 644 and 655.5 eV were associated with the +4 oxidation state (${Mn}^{+4}$) [32]. The presence of the +4 oxidation state in the Mn 2p spectrum may be related to the creation of photo-holes, which contribute to the change in its oxidation state, as described in Equation (5).

(5) $${Mn}^{+3}+h_{VB}^{+}\;\to \;{Mn}^{+4}\;$$

#### 3.6.4. Contribution of Reactive Oxygen Species to the Photodegradation of the MG Dye

**Figure 12:**
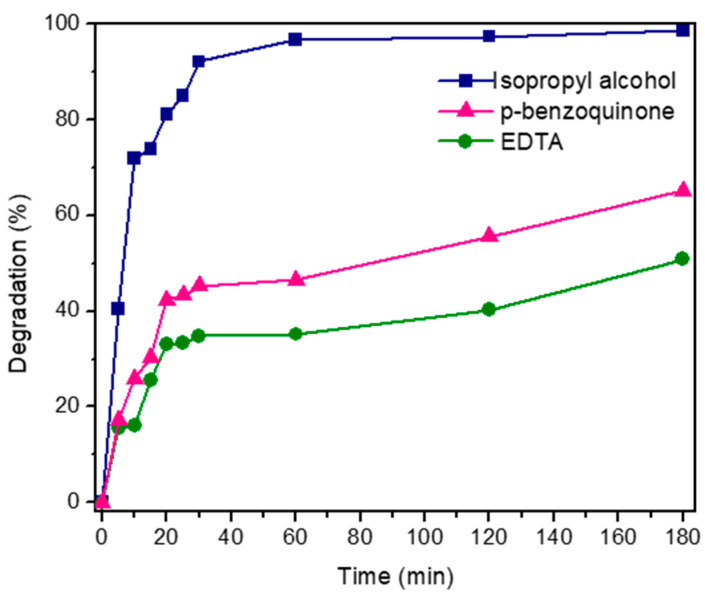
Percent degradation of MG dye using DyMnO_3_ as a photocatalyst and isopropyl alcohol, p-benzoquinone, and disodium ethylenediamine tetraacetic acid (EDTA) as scavengers.

To evaluate the contribution of reactive oxygen species (ROS) involved in the photocatalytic degradation process of MG dye, disodium ethylenediamine tetra acetic acid (EDTA), isopropyl alcohol (ISPA), and p-benzoquinone (p-BZQ) were used as scavengers of $h^{+}$, $OH^{•}$, and $O_{2}^{-},$ respectively. Three solutions with 40 mL of MG and 40 mg of DyMnO_3_ each were prepared for this. A scavenger (EDTA, ISPA, or p-BZQ) at a concentration of 0.5 × 10^−3^ M was added to each solution. The results are shown in Figure 12.

Comparing the photocatalytic efficiency of DyMnO_3_ obtained with each scavenger, it is observed that with EDTA, a photodegradation of 50% is achieved after 180 min. On the other hand, using p-BZQ and ISPA, 65% and 98% photodegradations are achieved within the same time interval, respectively. According to these results, holes ($h^{+}$) and hydroxyl radicals ($OH^{•}$) are the main ROS involved in the photocatalytic degradation process of MG dye. The superoxide radical ($O_{2}^{-}$) had the least contribution to the photodegradation of the dye.

#### 3.6.5. Photodegradation Mechanism

**Figure 13:**
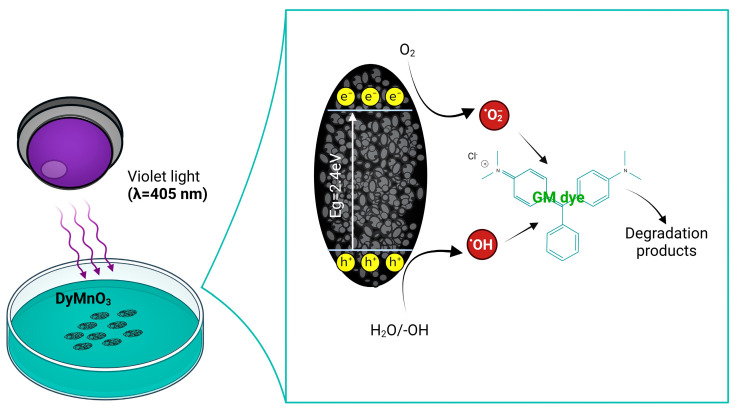
Graphical photodegradation mechanism of MG dye using DyMnO_3_ as photocatalyst under visible light (λ = 405 nm).

According to the results obtained from the scavengers and photocurrent tests, a possible photocatalytic degradation mechanism based on the formation of ROS was proposed (Figure 13) [33]. This mechanism can be explained as follows: Photons with energy hυ = 3.01 eV are emitted on the surface of DyMnO_3_ (*E_g_* = 2.40 eV). Since the energy of the incident photons, hυ, is greater than the *E_g_* of the oxide, electrons from the valence band (VB) can migrate to the conduction band (CB), forming electron–hole pairs (Equation (6)). The electrons in the conduction band promote the creation of superoxide radicals ($O_{2}^{-}$) (Equation (7)), which in turn contributes to the formation of hydroperoxyl ($OOH^{•}$) and hydroxyl (${OH}^{•}$) radicals (Equations (9)–(11)). On the other hand, the interaction of photo-holes ($h^{+}$) with water molecules and hydroxyl groups (−OH) also promotes the formation of ${OH}^{•}$ (Equations (8) and (12)). As it is known, ${OH}^{•}$ are strong oxidizing agents that can degrade organic molecules, in this case, allowing for the degradation of the MG dye, as shown in Equation (13).

(6) $$DyMnO_{3\left(surface\right)}\;+h\upsilon_{\left(photon\right)}\to e_{\left(CB\right)}^{-}+h_{\left(VB\right)}^{+}$$

(7) $$O_{2}+e_{\left(CB\right)}^{-}\to O_{2}^{-}$$

(8) $$H_{2}O+h_{\left(VB\right)}^{+}\to OH^{•}+H^{+}$$

(9) $$O_{2}^{-}+H^{+}\to {OOH}^{•}$$

(10) $${2(OOH}^{•})\to O_{2}+H_{2}O_{2}$$

(11) $$H_{2}O_{2}+O_{2}^{-}\to -OH+{OH}^{•}+O_{2}$$

(12) $$-OH+h_{VB}^{+}\to {OH}^{•}$$

(13) $${OH}^{•}+MG_{dye}\to Degradation\;products$$

### 3.7. Photocatalyst Oxides Used in the Degradation of MG Dye

Many oxides have been used as photocatalysts in the degradation of MG dye. Table 3 shows some of them as well as the conditions under which their photocatalytic activity was evaluated.

It is difficult to make a comparison regarding the photocatalytic efficiency of each one with that obtained with DyMnO_3_. This is because in many cases, the optical irradiance of the light-emitting source is not described, and in others, its photocatalytic activity was evaluated under basic or acidic conditions. Despite this, there are oxides that are more efficient than DyMnO_3_ in the degradation of this dye, such as Dy/ZnO or TiO_2_/WO_3_. However, they need to be mixed with other materials to achieve efficiencies greater than or similar to those obtained with dysprosium manganate.

## 4. Conclusions

DyMnO_3_ was synthesized using a novel and efficient polymer-decomposition method. Unlike other methods used in the synthesis of this oxide (such as hydrothermal, combustion, solid-state reactions), the method proposed in this work promotes the orthorhombic phase at 100 °C. Finally, at 800 °C, it was possible to obtain its orthorhombic phase without the presence of residual oxides (Mn_2_O_3_, Mn_3_O_4_, or Dy_2_O_3_). However, at 1000 and 1200 °C, diffraction lines associated with manganese oxides were observed.

Regarding its photocatalytic activity, a photodegradation of 80% of the MG dye was achieved in approximately 60 min under visible light (λ = 405 nm). The main reactive oxygen species (ROS) involved in the photodegradation process were $h^{+}$ and ${OH}^{•}$. Due to the conditions under which the photodegradation experiments were carried out (λ = 405 nm; *E_e_* = 100 mW/cm^2^), it is difficult to compare the efficiency obtained. However, under conditions similar to those described, the efficiency of this material is greater than that achieved with other oxides with a perovskite-type structure, particularly YMnO_3_, DyMnO_3_, and LaMnO_3_. A key factor associated with the photocatalytic efficiency of DyMnO_3_ was its reduced band gap energy (2.4 eV). This value allows us to approximate the minimum wavelength at which the separation of charge carriers ($h^{+}$, $e^{-}$) can occur in a photocatalyst. In this material, it was λ~516 nm.

In summary, there are diverse photocatalyst oxides, and among the most used are TiO_2_, ZnO, and CeO_2_. However, those oxides are very efficient under UV light (λ = 365 nm). Due to this, various synthesis methods have been developed that allow the band gap energy to be reduced. Moreover, they have been used in heterostructures in order to increase their photocatalytic activity at wavelengths in the visible spectrum. In this work, a new oxide photocatalyst with a perovskite-type structure (DyMnO_3_) was developed, with a degradation capability greater than 90% under visible light. However, its photocatalytic efficiency is lower than that of many heterostructures based on semiconductor oxides. For this reason, its use in heterostructures is considered future work with the purpose of further improving its photocatalytic efficiency.

## Figures and Tables

**Table 1:** Textural properties of DyMnO_3_ prepared by the polymer-decomposition method.

Material	SBET (m^2^ g^−1^)	BJH Total Pore Volume (cm^3^ g^−1^)	DR Micropore Volume (cm^3^ g^−1^)	BJH Pore Diameter (nm)
DyMnO_3_	7.3	0.0113	0.000128	2.4

**Table 2:** Some photocatalyst oxides used in the degradation of MG dye at *E_e_* = 100 mW/cm^2^.

Photocatalyst	Wavelength (nm)	Amount of Photocatalyst (mg)	Degradation Obtained after 60 min (%)
YMnO_3_	405	20	20 [7]
LaCoO_3_	365	10	50 [26]
DyCoO_3_	365	20	90 [27]
Ga_2_O_3_	365	30	10 [28]

**Table 3:** Photocatalyst oxides comparing the amount used, light type, irradiation time, and efficiency.

Photocatalyst	Photocatalyst Amount	Light Type	Irradiation Time (min)	Efficiency
ZnO and Dy/ZnO [33]	80 and 30 mg, respectively	Visible light	60	90% and 100%, respectively
Bi_2_WO_6_ [34]	1 g/L (pH = 2)	Visible light	30	87%
Fe_2_O_3_ (Hematite) [35]	0.1 g	UV	180	45%
NaNbO_3_ and Au/NaNbO_3_ [36]	0.4 g, respectively	Visible light	60	25% and 85%, respectively
WO_3_, TiO_2_/WO_3_ [37]	0.1 g, respectively	Visible light	60	50% and 100%, respectively
GO, CuFe_2_O_4_, GO/CuFe_2_O_4_ [38]	0.01 g, respectively	Visible light	210	90%, 30% and 63%, respectively
WO_3_ and Pd/WO_3_ [39]	150 mg/L, respectively	Solar light	300	50% and 80%, respectively
Bi_2_O_3_, CaFe_2_O_4_, B2O_3_/CaFe_2_O_4_ [40]	0.05 g, respectively	Visible light	240	70%, 58% and 89%, respectively
Fe_2_O_3_/SnO_2_ [41]	40 mg	Solar light	240	86%
ZnO [42]	0.2 g/L	Solar light	100	85%
Co_3_O_4_ [43]	50 mg	Visible light	100	90%
Cu_2_O [44]	10 mg	Visible light	45	92%
DyMnO_3_	40 mg	Visible light	60	93%

## Data Availability

The data presented in this study are available upon request from the corresponding author.
